# The Extraordinary Role of Extracellular RNA in Arteriogenesis, the Growth of Collateral Arteries

**DOI:** 10.3390/ijms20246177

**Published:** 2019-12-07

**Authors:** Anna-Kristina Kluever, Anna Braumandl, Silvia Fischer, Klaus T. Preissner, Elisabeth Deindl

**Affiliations:** 1 Walter-Brendel-Center of Experimental Medicine, University Hospital, Ludwig-Maximilians-University, 81377 Munich, Germany; A.Kluever@campus.lmu.de (A.-K.K.); Anna.Braumandl@med.uni-muenchen.de (A.B.); 2 Institute of Biochemistry, Medical School, Justus-Liebig-University, 35392 Giessen, Germany; Silvia.Fischer@biochemie.med.uni-giessen.de (S.F.); klaus.t.preissner@biochemie.med.uni-giessen.de (K.T.P.)

**Keywords:** arteriogenesis, VEGF, extracellular RNA, shear stress, endothelial activation, mast cell degranulation, macrophages, sterile inflammation, collateral artery growth, TACE

## Abstract

Arteriogenesis is an intricate process in which increased shear stress in pre-existing arteriolar collaterals induces blood vessel expansion, mediated via endothelial cell activation, leukocyte recruitment and subsequent endothelial and smooth muscle cell proliferation. Extracellular RNA (eRNA), released from stressed cells or damaged tissue under pathological conditions, has recently been discovered to be liberated from endothelial cells in response to increased shear stress and to promote collateral growth. Until now, eRNA has been shown to enhance coagulation and inflammation by inducing cytokine release, leukocyte recruitment, and endothelial permeability, the latter being mediated by vascular endothelial growth factor (VEGF) signaling. In the context of arteriogenesis, however, eRNA has emerged as a transmitter of shear stress into endothelial activation, mediating the sterile inflammatory process essential for collateral remodeling, whereby the stimulatory effects of eRNA on the VEGF signaling axis seem to be pivotal. In addition, eRNA might influence subsequent steps of the arteriogenesis cascade as well. This article provides a comprehensive overview of the beneficial effects of eRNA during arteriogenesis, laying the foundation for further exploration of the connection between the damaging and non-damaging effects of eRNA in the context of cardiovascular occlusive diseases and of sterile inflammation.

## 1. Introduction

Cardiovascular diseases such as ischemic heart disease, stroke or peripheral arterial occlusive disease are a major public health burden, accounting for approximately 30% of deaths worldwide in 2017 [1]. These diseases are commonly treated with percutaneous coronary interventions involving stents or with coronary bypass surgery. Interestingly enough, the body has a natural non-invasive way of forming a bypass around an occluded vessel called arteriogenesis. During arteriogenesis, blood flow is redirected through preexisting collateral arterioles upon occlusion of a supplying artery [2]. The main stimulus to initiate arteriogenesis in the pre-existing arteriolar vessels is increased fluid shear stress, which subsequently leads to endothelial cell activation, leukocyte extravasation and vessel wall (endothelial and smooth muscle cell) proliferation, substantially increasing the luminal diameter and restoring perfusion [2]. Whilst many of the steps leading to leukocyte extravasation and vessel growth have been uncovered, the crucial missing link of how intravascular shear stress is translated into local endothelial activation and vascular cell growth remained unknown.

Extracellular RNA (eRNA) released upon increased fluid shear stress during arteriogenesis in vivo has recently been suggested to be this missing link by initiating the cascade of arteriogenesis through vascular endothelial growth factor (VEGF)/VEGF receptor 2 (VEGFR2) signaling [3]. eRNA is released from cells upon cellular stress or damage and is mainly composed of rRNA [3,4]. Other forms of extracellular RNA such as microRNA have also been suggested to have a regulatory effect on collateral remodeling during arteriogenesis through modulation of intracellular signaling pathways; however, whether this effect is positive or negative seems to depend on the specific microRNA [5,6,7,8]. In terms of cardiovascular disease, eRNA released upon cellular damage has proven to have adverse effects in, e.g., ischemia/reperfusion injury, transplantation or atherosclerosis by mediating vascular edema, thrombus formation and inflammation [9,10,11,12,13,14]. This review aims to further elucidate the beneficial role of eRNA during the various stages of arteriogenesis.

## 2. The Role of eRNA in Arteriogenesis

### 2.1. eRNA Acts as a Translator of Shear Stress during Arteriogenesis through an Endothelial Mechanosensory Complex

**Figure 1:**
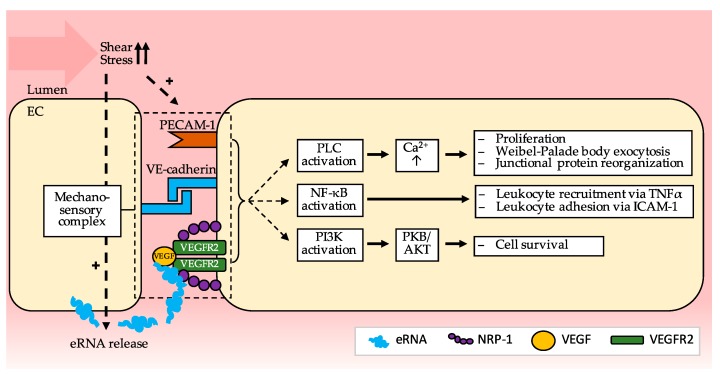
Proposed signaling mechanism downstream of the mechano-sensory complex composed of PECAM-1 (Platelet endothelial cell adhesion molecule (1), VE-cadherin (Vascular endothelial cadherin) and VEGFR2 (Vascular endothelial growth factor receptor (2) which could be relevant in arteriogenesis. eRNA released from EC (endothelial cells) upon shear stress enhances binding of VEGF to VEGFR2 and NRP-1 (Neuropilin-1) thus inducing the signaling mechanisms leading to endothelial cell activation and proliferation as well as leukocyte recruitment and adhesion. The intracellular compartment (EC) is depicted in yellow, the extracellular vessel lumen in red. Arrows indicate the various steps of the signaling pathways. [NF-κB (nuclear factor ’kappa-light-chain-enhancer’ of activated B-cells); PI3K (Phosphoinositide 3-kinases); PLC (Phospholipase C); PKB/AKT (Protein kinase B)].

The initiating stimulus for collateral remodeling in arteriogenesis is increased arteriolar fluid shear stress as a result of the occlusion of the main supplying artery [15]. In sharp contrast to other forms of vessel growth such as vasculogenesis or angiogenesis, vessel remodeling in arteriogenesis is stimulated by mechanical forces rather than by conditional factors such as hypoxia or ischemia [15,16]. Various mechanisms for shear stress sensing in endothelial cells have been described such as mechano-sensitive ion channels or the entire cytoskeleton transmitting changes in membrane tension (tensegrity architecture) [17]. However, it has recently been suggested that shear stress is in fact translated into endothelial cell activation through a mechanosensory complex, which was previously found to be located on endothelial cells in murine aortas predominantly at sites of non-laminar blood flow [18]. This complex comprises platelet endothelial cell adhesion molecule 1 (PECAM-1), vascular endothelial cell cadherin (VE-cadherin) and VEGFR2, whereby PECAM-1 acts as a mechano-sensor and together with VE-cadherin mediates VEGFR2 activation and subsequent intracellular signaling (Figure 1) [18]. VE-cadherin is an essential component of the endothelial adherens junction, mediating interactions with cytoskeletal anchoring molecules, and has been demonstrated to promote endothelial cell survival by enhancing VEGF-A signaling via VEGFR2 and subsequent phosphatidylinositol-3-OH-kinase activation as well as by activating protein kinase B (Akt) [19,20]. PECAM-1, also an adhesion molecule, has been shown to be involved in collateral remodeling in arteriogenesis as deficiency of PECAM-1 led to an attenuated increase in collateral luminal diameter and leukocyte recruitment to the perivascular space in a murine model of peripheral arteriogenesis [21]. Interestingly, in mice deficient in PECAM-1, the diameter of preexisting collaterals was larger than in wildtype mice; however, the number of preexisting collateral arterioles was comparable in both groups [21]. The signaling pathways activated by this mechanosensory complex that could also be highly relevant in arteriogenesis include (1) VEGFR2 activation, crucial for endothelial proliferation and von Willebrand factor (vWF) release, (2) nuclear factor κB (NFκB) activation, important for enhancing cytokine release and adhesion molecule expression, and (3) phosphatidylinositol-3-OH-kinase and protein kinase B (Akt) activation, essential for promoting endothelial cell survival [18].

#### 2.1.1. eRNA Initiates Arteriogenesis by Locally Enhancing VEGF/VEGFR2 Signaling

**Figure 2:**
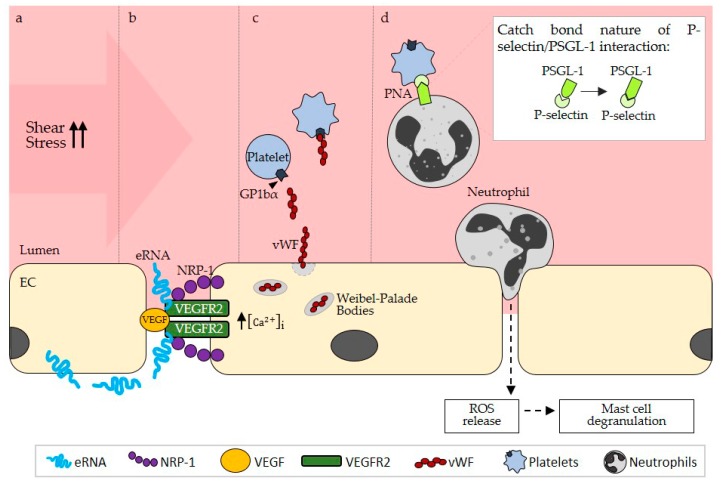
(**a**) Shear stress-induced activation of endothelial cells (EC) leads to the release of eRNA (extracellular RNA) on their abluminal side. (**b**) eRNA enhances the binding of VEGF (vascular endothelial growth factor) to NRP-1 (neuropilin (1) thus promoting VEGFR2 (VEGF Receptor (2) intracellular signaling. (**c**) VEGFR2 mediated raise of intracellular Ca^2+^ leads to the exocytosis of Weibel–Palade bodies and vWF (von Willebrand factor) release, which activates platelets via their GP1bα (Glycoprotein 1bα) receptor. (**d**) Upon activation, platelets express P-selectin, which subsequently binds its ligand PSGL-1 (P-selectin glycoprotein ligand-1) on neutrophils, inducing the formation of PNAs (platelet-neutrophil aggregates). The bond between P-selectin and PSGL-1 induces a deformational change, which promotes binding in these molecules (catch bond nature). PNA formation enhances neutrophil extravasation followed by ROS (reactive oxygen species) release which promotes mast cell degranulation. The intracellular compartment (EC) is depicted in yellow, the extracellular vessel lumen in red. Arrows indicate the subsequent steps during arteriogenesis.

VEGF is a glycoprotein produced by a variety of cell types including leukocytes such as neutrophils or monocytes [22,23,24,25] and is critically involved in enhancing endothelial cell proliferation, permeability, and angiogenesis [26,27,28]. The role of VEGF in arteriogenesis remained uncertain for a long time; however, it has now been established that its downstream signaling events are crucial for this process [3,25]. While it has been demonstrated that the expression of the isoform VEGF-A is not increased in collateral vessels during the process of arteriogenesis and that administration of additional VEGF does not significantly improve collateral vessel development [16,29], antibodies blocking either VEGF or the cognate receptor (VEGFR2) have been shown to interfere greatly with vessel remodeling [25,30,31]. This indicates that VEGFR2 signaling is relevant in arteriogenesis but that endogenous VEGF levels are sufficient for this process [25]. Under physiological conditions, the activation of VEGFR2 induces its dimerization and subsequent tyrosine kinase auto-phosphorylation and endocytosis, thus activating phospholipase C (PLC) and raising intracellular calcium levels [32]. These intracellular signaling events ultimately lead to endothelial activation including the release of Weibel–Palade bodies. Hereby, the co-receptor neuropilin-1 (NRP-1) plays an essential role in linking VEGF and its receptor and in enhancing subsequent intracellular signaling (Figure 2) [32]. In vitro and in vivo studies have demonstrated that NRP-1 is also relevant in arteriogenesis [33,34], whereby its cytoplasmic domain mediates both the co-endocytosis of NRP-1 and VEGFR2 as well as its interaction with synectin [32,33,34]. These results indicate that the VEGF/VEGFR2 system plays a part in arteriogenesis; nevertheless, they do not explain how VEGFR2 is locally activated in collaterals upon increased shear stress. This is where eRNA comes into play.

VEGFR2 activation and signaling have been demonstrated to be involved in mediating the pro-inflammatory and permeability-enhancing effects of eRNA on endothelial cells. Binding studies of eRNA to different VEGF isoforms (VEGF_165_ and VEGF_121_) confirmed that eRNA directly interacts with VEGF, most likely via its heparin binding domain [12]. Whilst eRNA does not alter the expression of either VEGF [12] or of VEGFR2 in vitro [35], it has been shown to enhance the binding of VEGF to NRP-1 in vitro [35]. The formation of this complex of eRNA with VEGF, NRP-1 and VEGFR2 thus promotes PLC-dependent intracellular signaling mechanisms. Since eRNA is released from endothelial cells upon shear stress in vitro [3], it was proposed that eRNA could also play a role in the early steps of arteriogenesis by activating the VEGF/VEGFR2 signaling pathway and thereby inducing endothelial activation. Accordingly, in a murine model of peripheral arteriogenesis, eRNA—mostly rRNA—was found to be released from endothelial cells of developing collaterals, which were exposed to increased shear stress, on their abluminal side [3]. The levels of VEGF bound to the glycocalyx of endothelial cells might be sufficient for initial local signaling induced by eRNA; however, as collateral remodeling progresses, leukocytes might take over the role of supplying VEGF [24,25]. Crucially, this shear stress-induced release of eRNA during arteriogenesis is not related to cellular injury, as no lactate dehydrogenase was released into cell supernatants in vitro [3]. In contrast, eRNA, which is involved in other pathological situations such as stroke, was liberated mostly as a result of tissue damage [9,12]. eRNA seems to play a stimulatory part in arteriogenesis as treatment of mice with RNA-degrading RNase1 led to a smaller increase in luminal diameter of the growing collateral vessels compared to saline-treated control mice and also impaired perfusion recovery in vivo [3,36]. In contrast, administration of DNase or inactive RNase1 did not alter perfusion recovery [3], supporting the notion that the observed effects of RNase1 resulted from the hydrolysis of eRNA rather than from other functions of the endonuclease. In accordance with this, the administration of an RNase inhibitor improved perfusion recovery as well [3], which is highly relevant since eRNA is rapidly degraded in the circulation by RNases under physiological conditions [37]. Therefore, eRNA released upon increased fluid shear stress seems to play a pivotal role in the early processes of arteriogenesis by mediating endothelial cell activation through an enhanced VEGF/NRP-1/VEGFR2 interaction and subsequent intracellular signaling.

#### 2.1.2. Contribution of eRNA in the Promotion of Collateral Remodeling through Stimulation of Endothelial NFκB Signaling

The extravasation of leukocytes, particularly of monocytes, is critical in arteriogenesis, since the cytokines and growth factors released by these cells are essential stimulators of endothelial and smooth muscle cell proliferation [38,39]. eRNA has previously been established as a strong promoter of leukocyte adhesion and extravasation, especially of monocytes, as demonstrated in a murine cremaster model and a murine model of atherosclerosis [11,14]. This pro-inflammatory effect was also confirmed in the process of arteriogenesis [3], where RNase1 treatment reduced the extravasation of neutrophils and monocytes in vivo [3]. In this case, no change in blood levels of leukocytes was observed [3], indicating that eRNA affects local cell interactions but not the systemic cell count. Physiologically and in arteriogenesis, leukocyte transmigration is facilitated by the interaction between endothelial adhesion molecules such as intercellular adhesion molecule 1 (ICAM-1) and leukocyte ligands such as macrophage-1 antigen (Mac-1) [40,41]. The expression of ICAM-1 has independently been shown to be upregulated on the one hand by fluid shear stress in vitro and in vivo [42,43] and on the other hand by eRNA in vitro [14,44]. In an in vivo model of peripheral arteriogenesis, the effects of eRNA on endothelial cell signaling were also demonstrated to be relevant for subsequent leukocyte adhesion, as inhibition of eRNA through RNase administration reduced the perivascular macrophage count to a similar degree as seen in ICAM-1 deficient mice [3].

Two possible ways through which eRNA might affect the expression of ICAM-1 are through stimulation of the NFκB or of the VEGFR2 signaling pathways. VEGF has been shown to induce the upregulation of ICAM-1 in a transgenic mouse model of psoriasis [45], whereby its effects on ICAM-1 expression were demonstrated to be the result of an enhanced VEGF/NRP-1 interaction in a retinal mouse model [46]. Since eRNA enhances the formation of an activated VEGF/NRP-1/VEGFR2 complex during arteriogenesis, it might promote the expression of adhesion molecules on endothelial cells through this avenue of signaling. Nevertheless, eRNA has also been shown to promote the activity of NFκB, which also enhances ICAM-1 expression [47], by inducing the phosphorylation of the cytoplasmic inhibitor of κB (IκB) in vitro [14]. The role of NFκB signaling in arteriogenesis has not yet been studied in detail; however, this pathway was found to be continuously activated in situations of disturbed blood flow such as in atherosclerosis [18]. Furthermore, an increase in the nuclear translocation of NFκB and hence an increase in the expression of its target genes was observed in a rat mesenteric model of collateral growth [48]. Moreover, NFκB is also able to induce the expression of VEGF [49], suggesting that there might be an interplay between these two avenues of signaling in mediating the effects of eRNA on endothelial activation during arteriogenesis.

### 2.2. eRNA Is Relevant for Mast Cell Degranulation during Arteriogenesis by Stimulating vWF Release and PNA Formation

#### 2.2.1. eRNA Mediates vWF Release from Endothelial Weibel–Palade Bodies by Promoting VEGF/ VEGFR2 Signaling

Through its stimulatory effect on VEGF/VEGFR2 signaling, eRNA mediates endothelial activation and the release of vWF from Weibel–Palade bodies, which is essential for subsequent platelet activation and platelet-neutrophil aggregate (PNA) formation in arteriogenesis (Figure 2). Endothelial cell activation as seen in arteriogenesis is characterized to a large extent by a loss of vascular barrier function, luminal expression of adhesion molecules for leukocytes and release of cytokines and pro-coagulatory molecules [50]. eRNA has been associated with all of these events, since it mediates hyper-permeability and vascular edema formation under conditions of ischemia/reperfusion injury or transplantation by disrupting endothelial tight junctions and since it promotes thrombus formation by activating the contact phase system of intrinsic coagulation [4,10,12,13]. In the inflammatory setting of arteriogenesis where eRNA plays such a prominent role, this raises the question of how thrombus formation is inhibited, which will be discussed at a later stage of this review. In addition to these pro-inflammatory and pro-coagulatory effects, eRNA also stimulates the exocytosis of endothelial cell storage granules, designated as Weibel–Palade bodies, which contain vWF, P-Selectin and other cytokines [51,52,53], as indicated by increased vWF release from endothelial cells upon eRNA exposure in vitro [35]. The exocytosis of Weibel–Palade bodies can also be induced by various other stimuli including thrombin, VEGF and reactive oxygen species (ROS) [54,55,56]. Weibel–Palade body exocytosis by eRNA was inhibited by an antibody against VEGF [35], suggesting that eRNA stimulates vWF release from endothelial cells via VEGF/VEGFR2 signaling as described above [12]. In the context of arteriogenesis, the ability of eRNA to induce endothelial activation and vWF release via VEGFR2 activation was confirmed, since RNase1 treatment in a murine model of peripheral arteriogenesis decreased luminal vWF levels to a similar extent as seen with vWF deficiency or VEGFR2 inhibition [3].

#### 2.2.2. eRNA-Mediated vWF Release Is Pivotal for PNA Formation

vWF is an adhesive and prothrombotic glycoprotein, constitutively produced by endothelial cells and released from endothelial Weibel–Palade bodies upon cell stimulation. It forms large and ultralarge multimers and plays a key role in the tethering and adhesion of platelets to the injured vessel wall, particularly under fluid shear stress conditions [57,58]. In arteriogenesis, the release of vWF and its interaction with platelets have been demonstrated as pivotal, triggering the activation of platelets and the formation of PNAs (Figure 2) [59,60]. PNAs in turn have been implicated in the recruitment and priming of neutrophils during inflammatory processes and were shown to be relevant for the release of reactive oxygen species (ROS) [61], both of which are relevant in arteriogenesis [60,62]. Following neutrophil extravasation and ROS release, perivascular mast cells are activated and the subsequent extravasation of leukocytes and their release of cytokines and growth factors are increased. In this way, the proliferation of endothelial cells and smooth muscle cells is stimulated, collateral vessels are able to expand, and perfusion can be sufficiently restored. PNAs are also crucial for the formation of neutrophil extracellular traps (NETs), comprising the entire decondensated chromatin of neutrophils, whose formation has also been induced following stimulation of neutrophils with extracellular nucleic acids [63]. In the context of arteriogenesis, though, the formation of NETs has not yet been described. However, since the application of DNase had no effect on perfusion recovery in vivo [3], one can assume that even if NETs are formed during arteriogenesis, they would not have a negative effect on vessel remodeling.

While free platelets do not usually interact with vWF, they are able to transiently bind to vWF via their receptor glycoprotein Ibα (GPIbα) under high fluid shear stress, resulting in platelet activation and adhesion [64]. GPIbα also binds to other pro-coagulatory factors such as thrombin or factors XI and FXII as well as to adhesion molecules such as P-selectin or Mac-1 on immune cells, thereby facilitating cell-cell contacts [65]. Interestingly, the vWF-GPIbα interaction appears to be optimal at high fluid shear stress, a condition which is predominantly found in arterioles [64,65,66], which is also where vessel remodeling takes places during arteriogenesis. This seems to be due to the fact that shear stress induces conformational changes in the vWF multimers [64], which then facilitate their interactions with platelets. These interactions are also vital in the high shear stress setting of arteriogenesis, where both platelet deficiency and GPIbα deficiency resulted in reduced perfusion recovery in vivo [59]. Once platelets are activated through vWF/GPIbα binding, they express P-selectin on their surface, which is subsequently able to bind to the corresponding P-selectin glycoprotein ligand-1 (PSGL-1) on neutrophils [67,68], enabling the formation of PNAs [60]. The expression of P-selectin has been demonstrated to be increased by shear stress as well [67], and additionally, it was suggested that the resulting adhesive bonds between P-selectin and PSGL-1 are stronger under high fluid shear stress owing to the catch bond nature of selectins [40]. P-selectin and PSGL-1 seem to mediate the interactions of platelets with neutrophils at higher rates of shear stress in vitro, while integrin-mediated interactions seem to be more relevant at lower levels of fluid shear stress [69]. The formation of PNAs was also observed in vivo in the high fluid shear stress setting of arteriogenesis, whereby this binding of platelets to neutrophils was abolished in GPIbα knock-out mice [59], underlining the relevance of vWF release and binding to GPIbα for PNA formation in arteriogenesis.

This important step during collateral remodeling might also be influenced indirectly by eRNA, marking another point where eRNA could play a role during arteriogenesis. In a murine model of arteriogenesis, RNase1 treatment decreased the formation of PNAs to a similar extent as was seen in mice which were either deficient in vWF or treated with a VEGFR2 inhibitor [3], suggesting that VEGF/VEGFR2-dependent eRNA-mediated effects on vWF release also ultimately affect PNA formation. Critically, the administration of RNase1 did not change the blood levels of platelets or neutrophils [3], indicating that eRNA-mediated effects are not systemic but localized to collaterals and do not involve enhanced release of effector cells from the bone marrow. In addition, activated platelets are able to trigger the expression of P-selectin on endothelial cells, thus enabling leukocyte rolling along the activated vessel wall [70]. Such transient interactions of platelets with endothelial cells were also observed in arteriogenesis [59]. In summary, the release of vWF from endothelial Weibel–Palade bodies is a critical step in arteriogenesis as vWF subsequently leads to the activation of platelets and the formation of PNAs, and these processes depend on eRNA, which enhances VEGF/VEGFR2 signaling and endothelial activation.

#### 2.2.3. The Possible Role of ADAMTS13 in the Suppression of Thrombus Formation in Arteriogenesis

The activation of platelets together with the secretion of highly reactive multimeric vWF from endothelial cells under inflammatory conditions during the process of arteriogenesis would provide an ideal setting for thrombus formation. Yet, such an outcome has never been observed, and it remains to be studied in which way local thrombus formation is inhibited during arteriogenesis. Following activation of endothelial cells and the initial release of vWF in a multimeric form to promote platelet adhesion under flow, the readily coagulable nature of these large vWF multimers needs to be weakened at a later phase to prevent the formation of microthrombi [71]. vWF can be cleaved via limited proteolysis by the circulating plasma metalloprotease ADAMTS13 (a disintegrin and metalloprotease with a thrombospondin type 1 motif, member 13) [57]. The deficiency of this protease has been associated with a thrombotic pathology known as thrombotic thrombocytopenic purpura [72]. The interaction between vWF and ADAMTS13 was revealed to be increased under high shear stress in vitro and was maintained even after shear stress was eliminated [58,73]. This effect has been attributed to unfolding of the vWF molecule from a globular to an elongated form upon exposure to shear stress and subsequent exposition of scissile bonds for cleavage by ADAMTS13 [57]. Cleavage of vWF by ADAMTS13 could therefore be a potential mechanism through which thrombus formation could be inhibited in arteriogenesis. In fact, platelet adhesion and aggregation reactions were increased in the arterioles of ADAMTS13-deficient mice compared to wild-type mice; however, this situation could be reversed by the administration of recombinant ADAMTS13 [74]. Furthermore, in a murine model of stroke, ADAMTS13 deficiency was associated with impaired angiogenesis and brain capillary perfusion [75]. The pro-angiogenic properties of ADAMTS13 have been proposed to be due to the phosphorylation of VEGFR2 and upregulation of VEGF, both in vivo and in vitro [75,76], which was also reflected by the fact that an in vitro siRNA-induced knockdown of ADAMTS13 reduced VEGF levels in the cell lysate as well as endothelial proliferation [77]. ADAMTS13 therefore seems to influence VEGF signaling, and since the effect of eRNA on this signaling pathway has proven to be pivotal in arteriogenesis, there might be a link between eRNA and ADAMTS13 in arteriogenesis as well. Yet, the exact role of ADAMTS13 during this process still needs to be assessed.

#### 2.2.4. eRNA is Relevant for Mast Cell Degranulation Following PNA Formation

**Figure 3:**
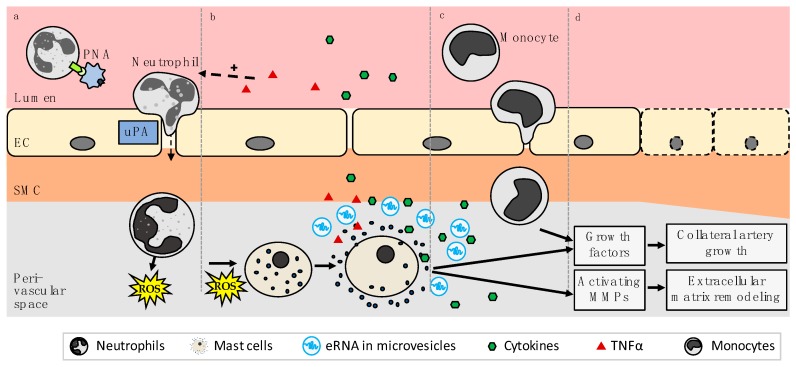
(**a**) PNA (platelet neutrophil aggregate) formation leads to uPA (Urokinase-type plasminogen activator) mediated neutrophil extravasation and subsequent ROS (reactive oxygen species) release. (**b**) ROS, released from neutrophils, initiates mast cell degranulation and consecutive release of cytokines, growth factors and eRNA in microvesicles. (**c**) eRNA and cytokine (especially MCP-1) mediated recruitment and extravasation of monocytes enhances local inflammatory processes leading to (**d**) collateral artery growth (arteriogenesis) and extracellular matrix remodeling. The extracellular vessel lumen is depicted in red, EC in yellow, SMC in orange, and the perivascular space in gray. Arrows indicate the subsequent steps during arteriogenesis. [EC: endothelial cells; SMC: smooth muscle cells].

Following PNA formation, the extravasation of neutrophils is mediated through urokinase-type plasminogen activator (uPA) [60], which, in contrast to tissue plasminogen activator, has been shown to be vital for the transmigration of leukocytes during arteriogenesis in vivo [62]. The subsequent release of NADPH oxidase 2 (Nox2)-derived ROS from neutrophils is crucial for inducing mast cell degranulation [60], whereby neutrophils in PNAs were shown to produce more ROS than non-complexed neutrophils [61]. Both the surface expression of uPA as well as ROS formation via Nox2 were reduced in P-selectin-deficient and PSGL-1-deficient cells in vitro [60], underlining the importance of PNAs in triggering the steps leading to mast cell degranulation in arteriogenesis. Perivascular mast cells in turn have been established as key players during collateral remodeling and have been suggested to act in three ways: (1) by releasing cytokines, which promote the recruitment of growth factor producing leukocytes, (2) by themselves supplying growth factors for the growing collateral, and (3) by activating matrix metalloproteases (MMPs), responsible for extracellular matrix remodeling (Figure 3) [60]. Mast cell degranulation in arteriogenesis has also been shown to be promoted following eRNA stimulation of endothelial VEGF/VEGFR2 signaling, as RNase1 administration impaired mast cell degranulation in an in vivo model of arteriogenesis to a comparable degree as VEGFR2 inhibition and vWF deficiency did without affecting the perivascular mast cell count [3].

### 2.3. eRNA Released from Mast Cells Provides a Second Stimulatory Boost for Collateral Remodeling

As previously described, eRNA released by endothelial cells upon cellular stress such as fluid shear stress has been attributed an essential role as the translator of shear stress into endothelial activation during the initial stages of collateral remodeling. However, eRNA is rapidly degraded by circulating RNases and could thus only unfold its actions over a short period of time [37]. Arteriogenesis, on the contrary, is a chronic process. Mast cells, whose degranulation is stimulated by the downstream effects of endothelial cell-derived eRNA, also release eRNA in the form of microvesicles concomitantly with degranulation [44]. This release of eRNA has been shown to promote local inflammatory processes and leukocyte extravasation [44]. Microvesicles (MV) are a type of extracellular vesicles that have been described to be released from a variety of cell types including endothelial cells, leukocytes, platelets and mast cells [78,79,80,81], often upon cell stress [82], to be involved in cell communication and inflammation including during angiogenesis [82,83], and to be selectively enriched with different types of RNA [84]. Microvesicles/microparticles are generated via budding from the cell membrane and can reach a diameter of up to 1000 nm, whereas exosomes, another type of extracellular vesicle, are released via exocytosis and are generally smaller than 100 nm [82]. In the context of arteriogenesis, this additional release of eRNA in microvesicles might act as a booster of the initial effects mediated by endothelial cell-derived eRNA. What remains to be determined is the exact mechanism through which mast cell-derived eRNA unfolds its effects. However, it is plausible that it also stimulates VEGF/VEGFR2 signaling in endothelial cells. This is supported by the fact that various leukocytes including neutrophils and monocytes, whose extravasation is critical in arteriogenesis, secrete VEGF and have been demonstrated to do so in arteriogenesis [22,23,24,25]. In addition, treatment of endothelial cells with MVs derived from mast cells potentiated the release of vWF from endothelial cells just as endothelial cell-derived eRNA did [44]. Besides acting as a positive feedback mechanism for endothelial activation, an important effect of mast cell-derived eRNA might also be the stimulation of the expression of adhesion molecules on endothelial cells as was shown for ICAM-1 in vitro [44]—potentially through VEGFR2 or NFκB signaling.

Furthermore, eRNA released by mast cells might also directly influence leukocyte adhesion, transmigration and cytokine/chemokine release such as of TNFα or MCP-1 during arteriogenesis and might thus amplify the budding local inflammatory response. TNFα is a key pro-inflammatory cytokine released by a variety of cell types including granulocytes, macrophages and smooth muscle cells and is able to enhance leukocyte adhesion, coagulation, and endothelial permeability [85,86,87]. Its ability to promote MCP-1 expression and monocyte extravasation are extremely relevant in arteriogenesis [88], as is its stimulation of neutrophil and T-cell extravasation [60,89,90,91]. In a murine model of peripheral arteriogenesis, the inhibition of TNFα lead to reduced perfusion recovery and, in a rabbit model, to a decrease in luminal vessel diameter, smooth muscle cell proliferation and leukocyte extravasation [92,93]. MCP-1 has been established as a major trigger of monocyte recruitment and accelerator of collateral growth during arteriogenesis [94,95,96,97], and key sources of this chemokine include macrophages, endothelial cells and smooth muscle cells [98,99,100]. eRNA was found to promote the release of TNFα from monocytes by activating the TNFα converting enzyme (TACE/ADAM17), which releases soluble TNFα from its membrane-bound form [101]. The inhibition of TACE reduced both the eRNA-mediated release of TNFα in vitro and the adhesion of leukocytes following eRNA administration in vivo [14]. This effect of eRNA on TACE activation in macrophages in vitro was found to involve NFκB signaling [102]. The effects of eRNA on TACE activity in arteriogenesis have not yet been explored; however, it would be interesting to see if eRNA released by mast cells could stimulate the release of TNFα via TACE and thus boost the local inflammatory response. Nevertheless, both TNFα and eRNA were demonstrated to have similar effects on cell adhesion to the endothelium in murine cremaster venules in the context of arteriogenesis [3,14]. The stimulatory effect of eRNA on cell adhesion was abolished by pre-treatment with a VEGFR2 inhibitor (Semaxanib), again highlighting that eRNA enhances VEGFR2 activation, whereas the TNFα-mediated effect was not diminished by pre-treatment with Semaxanib [3], since the release of TNFα occurs at a later stage of arteriogenesis following VEGFR2 activation and signaling. The expression of MCP-1, on the other hand, was increased by MV-derived eRNA from mast cells in vitro [44]. eRNA released by mast cells might therefore activate TACE and thus stimulate TNFα and subsequent MCP-1 release in arteriogenesis, thereby enhancing the local inflammatory response and leukocyte transmigration. In addition, NFκB, whose activation can be stimulated by eRNA as previously touched upon, can also induce the expression of pro-inflammatory cytokines such as TNFα [103], suggesting that mast cell-derived eRNA might also promote local inflammation through this avenue of signaling. However, the exact ways through which this second release of eRNA enhances leukocyte extravasation and collateral remodeling during arteriogenesis still have to be confirmed.

At this point, it is interesting to note that eRNA administration in vitro was shown to prompt a shift in macrophage polarization towards the pro-inflammatory M1 phenotype with an upregulation of pro-inflammatory cytokines such as TNFα and a concurrent downregulation of anti-inflammatory cytokines [104]. In the context of arteriogenesis, the pro-inflammatory M1 macrophage phenotype is important during the initial stages of vessel remodeling, which would correlate with the release of eRNA during arteriogenesis and would suggest that the previously described eRNA-induced shift in macrophage polarization could also occur during arteriogenesis, whilst the anti-inflammatory M2 phenotype is more relevant during later stages of vessel remodeling [105].

## 3. eRNA in Other Forms of Vessel Growth

The role of eRNA in other forms of vessel growth, namely vasculogenesis and angiogenesis, has not been studied in great detail so far. In vasculogenesis, tRNA and rRNA were found to stimulate the formation of new vessels and the leukocyte differentiation of embryonic bodies via increased VEGF signaling and ROS generation [106]. VEGF signaling is crucial in vasculogenesis [107], and upon eRNA exposure, the expression of VEGF_165_, NRP-1 and other hypoxia related factors such as HIF-1α was increased [106]. ROS formation was also enhanced after eRNA administration [106], which is significant, since the interplay between intracellular VEGF signaling and ROS generation is a central issue of embryoid body differentiation [108]. In angiogenesis, the focus has been more on the stimulatory role of endogenous RNases namely of angiogenin, also known as hRNase5, as a strong promoter of endothelial cell proliferation through its effects on rRNA and ribosome synthesis and its regulatory role in translation during cellular stress [109,110].

## 4. Conclusions

The remodeling of pre-existing arteriolar collaterals during arteriogenesis is a complex process requiring the highly-coordinated interplay of different leukocytes to promote endothelial and smooth muscle cell proliferation and to ultimately establish perfusion. eRNA has been demonstrated to translate fluid shear stress into endothelial activation during arteriogenesis, further stimulating vWF release, PNA formation, mast cell degranulation and leukocyte recruitment, culminating in the beneficial process of arteriogenesis to promote perfusion recovery. In contrast to the beneficial role of eRNA in arteriogenesis, eRNA has been formerly established as a damaging or pathological factor in a variety of cardiovascular diseases based upon its modulation of endothelial cell and leukocyte function, whereby administration of RNase1 was demonstrated to serve as a tissue- and vessel-protective regimen. It remains to be established which particular molecular interactions and binding partners as well as which putative cellular receptors for eRNA appear to be responsible for its either adverse or beneficial functions in the cardiovascular system.

## Abbreviations

ADAMTS13	A disintegrin and metalloprotease with a thrombospondin type 1 motif, member 13
eRNA	Extracellular RNA
ICAM-1	Intercellular adhesion molecule 1
Mac-1	Macrophage-1 antigen
MCP-1	Monocyte chemoattractant protein 1
MMP	Matrix metalloprotease
MV	Microvesicle
NFkB	Nuclear factor kB
Nox2	NADPH oxidase 2
NRP-1	Neuropilin-1
PECAM-1	Platelet endothelial cell adhesion molecule 1
PLC	Phospholipase C
PNA	Platelet-neutrophil aggregate
ROS	Reactive oxygen species
TACE	TNFa converting enzyme
TNFa	Tumor necrosis factor a
uPA	Urokinase-type plasminogen activator
VE-cadherin	Vascular endothelial cell cadherin
VEGF	Vascular endothelial growth factor
VEGFR2	Vascular endothelial growth factor receptor 2
vWF	Von Willebrand factor
